# Pesticide use and opportunities of exposure among farmers and their families: cross-sectional studies 1998-2006 from Hebron governorate, occupied Palestinian territory

**DOI:** 10.1186/1476-069X-9-63

**Published:** 2010-10-19

**Authors:** Yaser Issa, Farid Abu Sham'a, Khaldoun Nijem, Espen Bjertness, Petter Kristensen

**Affiliations:** 1 University of Oslo, Faculty of Medicine, Institute of Health and Society, Department of General Practice and Community Medicine, Section for Preventive Medicine and Epidemiology; 2 Occupational Epidemiology and Biological Research Lab, Department of Biology, Hebron University, occupied Palestinian territory; 3 Environmental Health Department, Ministry of Health, occupied Palestinian territory; 4 Hebron Governmental Hospital, Ear Nose Throat Department, Hebron, West Bank, occupied Palestinian territory; 5 Tibet University Medical College, Lhasa, Tibet, China; 6 Department of Occupational Medicine and Epidemiology, National Institute of Occupational Health, Oslo, Norway

## Abstract

**Background:**

Adverse health effects caused by pesticide exposure have been reported in occupied Palestinian territory and the world at large. The objective of this paper is to compare patterns of pesticide use in Beit-U'mmar village, West Bank, between 1998 and 2006.

**Methods:**

We studied two populations in Beit-U'mmar village, comprised of: 1) 61 male farmers and their wives in 1998 and 2) 250 male farmers in 2006. Both populations completed a structured interview, which included questions about socio-demographic factors, types of farming tasks, as well as compounds, quantities, and handling of pesticides. Using the 1998 population as a reference, we applied generalized linear regression models (GLM) and 95% confidence intervals (CI) in order to estimate prevalence differences (PD) between the two populations.

**Results:**

In 1998, farmers used 47 formulated pesticides on their crops. In 2006, 16 of these pesticides were still in use, including five internationally banned compounds. There were positive changes with less use of large quantities of pesticides (>40 units/year) (PD -51; CI -0.60, -0.43), in applying the recommended dosage of pesticides (PD +0.57; CI +0.48, +0.68) and complying with the safety period (PD +0.89; CI+0.83, +0.95). Changes also included farmers' habits while applying pesticides, such as less smoking (PD -0.20; CI-0.34, -0.07) and eating at the work place (PD -0.33; CI-0.47, -0.19). No significant changes were found from 1998 to 2006 regarding use of personal protective equipment, pesticide storage, farmers' habits after applying pesticides, and in using some highly hazardous pesticides.

**Conclusions:**

The results were based on two cross-sectional surveys and should be interpreted with caution due to potential validity problems. The results of the study suggest some positive changes in the handling of pesticides amongst participants in 2006, which could be due to different policy interventions and regulations that were implemented after 1998. However, farm workers in Beit -U'mmar village are still at risk of health effects because of ongoing exposure to pesticides. To the best of our knowledge, no studies on long-term changes in pesticide use have been reported from developing countries.

## Background

Agricultural workers are at risk of exposure to occupational hazardous factors, including pesticides, dust, bacteria, moulds, endotoxins, and ammonia [1]. Pesticides are chemical substances used to protect agricultural crops and they have helped limit and control the spread of certain human diseases, such as malaria. However, pesticides also endanger humans and the environment [2]. Toxic, environmentally persistent, and inexpensive chemicals are used intensively in developing countries [3], which make up about 20% of the world pesticide usage [4]. The use of pesticides has increased due to widespread application in agricultural and environmental pest control [5].

Many farming activities pose serious risks of pesticide exposure, such as land preparation for cultivation, storing, mixing, preparing and spraying of pesticides, and loading and cleaning of spraying equipment [6]. Exposure to pesticides may result in acute and chronic health problems, including temporary acute effects like irritation of eyes and excessive salivation, as well as chronic diseases like cancer and reproductive and developmental disorders [7]. Others have reported adverse health effects, such as dermatitis, asthma [8], peripheral nerve effects, chronic neurobehavioral, motor dysfunction [9,10], burning sensations in eyes/face, skin irritation, headache, dizziness, and respiratory effects [11-15]. Acute poisoning, toxicity, and death have also been reported among farmers from occupied Palestinian territory (oPt) and other parts of the world [12,16-19].

In oPt, agriculture contributes to 33% and 24% of the Gross National Products (GNP) in the West Bank and Gaza Strip, respectively [20]. It is estimated that 96.6% of irrigated land and 87.0% of rain-fed land are treated with pesticides [20]. In the Gaza Strip, it is estimated that the annual use of pesticides, including some pesticides which have been suspended or banned for use in different parts of the world [12,18-21], exceeds 250 metric tonnes, in addition to 1000 metric tonnes of methyl bromide. In the West Bank in 1995, 123 different pesticide compounds with a total quantity of approximately 730 tonnes were used [22].

In oPt, there has been a reduction in the use of pesticides from 1388 tonnes in 2005 to 977 tonnes in 2006 [23]. Pesticides are purchased in Israel and distributed to Palestinian farmers through merchants and pesticide distributors at the Palestinian markets [11,20,24]. However, illegal pesticides are still in use in oPt. Further, Palestinian farmers and household members are at risk to be exposed to pesticides due to improper application, storing, and disposal [11,20,22,25]. Palestinian farmers have tended to use pesticides in higher amounts than necessary; they rarely used personal protective equipments (PPE); they prepared (mixed) and stored pesticides in their homes; and they cleaned the equipment at home [11,24].

The aim of this paper is to explore possible differences in farmers' use of pesticides (compounds, quantities, and handling) and PPE in Beit-U'mmar village in 1998 and 2006.

## Methods

### Study area

The two studies were undertaken in Beit-U'mmar village, which is one of the largest agricultural areas in oPt's Hebron district [Agricultural department of Hebron, 2005, personal communication]. According to the municipality data, about 22,300 dunums (1 dunum = 1000 square meter) of land in Beit-U'mmar are used for agriculture. Nearly 60% of the population works in the agriculture sector, all of whom are farm owners. Of the remaining 40% of the population, 13% are employees, 15% work in Israel, 2% work in the service sector, 2% work in the industrial sector, and 8% work in the trade sector. The main crops cultivated by farmers in both studies were grapes, plums, vegetables and apples [Beit-U'mmar municipality, 2006, personal communication]. In Beit-U'mmar, farming is a family business, with all family members being engaged in agricultural activities, Hence, even children of these families appear to be at risk of pesticide exposure. Furthermore, the total population of the village is about 14,000 inhabitants, of whom 45.4% are less than 15 years old [26].

### Design

A cross-sectional study design was adopted for both studies in which 61 farming couples (1998) and 250 male farmers (2006) were selected to participate in a semi-structured interview. The 1998 survey was a preliminary study in order to examine pesticide use and its relation to farmer families' reproductive functions. The 2006 was part of a study investigating the relation between pesticides and respiratory function.

### Study population

In 1998, there was no official registration of farmers in the village municipality; therefore, names of farmers were obtained from a list prepared by the central markets where farmers sent their products. Selected cases included couples with at least one child or where the woman was pregnant; the farmer owned at least five dunums of cultivated land; and the farmer had been working in agriculture for at least 5 years. At the beginning we intended to include all 208 registered farmers that fulfilled this criteria, but only 61 couples accepted to participate in the study (response rate 61/208 = 29.3%).

In the 2006 study, most farmers had been registered in the municipality records. We selected 250 male farmers alphabetically from the list of the 3000 registered farmers. Because ten farmers (4.0%) had refused to participate (response rate of 96.2%.), we invited the next 10 farmers on the list to reach the goal of 250 participants. Nine farmers participated both in 1998 and 2006.

### Questionnaires

Two slightly different questionnaires were used in the two periods. Both questionnaires included questions about socio-demographic factors, such as age, educational level, smoking habits, number of children, and socio-economic status defined according to farm size (low = ≤ 1ha', medium = 1.1-2.0 ha', and high = >2 ha') (Table 1). They also included identical questions on agricultural and farming activities, such as the number of years working in agriculture; the types of farming tasks performed; the number of years farmers had been using pesticides; the total quantity of pesticides applied per year; the frequency with which pesticides were applied annually; the length of time that pesticides were applied per hour/year; the type of spraying equipment used; and the type of cultivated crops where pesticides were used (Table 2), Also, the names of the most commonly used pesticides were provided (Table 3). In addition, both the 1998 and the 2006 questionnaire included items concerning the handling and practicing of pesticides. Questions concerned the required or needed amounts of pesticides on the farm; whether pesticides were prepared, mixed, and stored in the home; if work clothes were washed together with family clothes; if spraying equipment was cleaned after use in the orchards; if the farmer used to eat or smoke at the workplace; and if the safety period of applied pesticides was followed (Table 4). Finally, questions regarding the use of personal protective equipment (PPE) were included (Table 5). We considered the 1998 questionnaire to be base-line, and all identical questions on the two occasions were compared.

**Table 1:** Socio-demographic characteristics of participating farmers in Beit-U'mmar village, 1998 and 2006.^a^

	1998 (n = 61 couples)				2006 (n = 250 male farmers)	
		
	Husbands		Wives			
	
Characteristic	**No**.	(%)	**No**.	(%)	**No**.	(%)
**Age in years**						

20-29	4	(7.0)	14	(23.0)	27	(10.8)

30-39	17	(28.0)	18	(30.0)	68	(7.2)

40-49	17	(28.0)	20	(33.0)	53	(21.2)

50-59	19	(31.1)	8	(13.0)	44	(17.6)

60-69	4	(7.0)	1	(2.0)	41	(16.4)

>69	0.0	(0.0)	0.0	(0.0)	17	(6.8)

Mean (SD)	44.9	(11.0)	39.0	(11.0)	46.6	(14.4)

**Education in years**						

1-10	28	(46.0)	50	(82.0)	121	(48.4)

11-12	12	(20.0)	6	(10.0)	56	(22.4)

>12	21	(34.4)	5	(8.0)	73	(29.2)

Mean (SD)	10	(4.0)	8	(3.2)	10	(4.3)

**Smoking**						

Yes	49	(80.3)	5	(8.2)	91	(36.4)

No	12	(19.7)	56	(91.8)	159	(63.6)

**No. of children**						

0-4	22	(36.0)			95	(38.0)

5-9	32	(53.0)			114	(45.6)

>9	7	(12.0)			41	(16.4)

Mean (SD)	7	(2.3)			6	(4.0)

**Socio-economic status**^**b**^						

Low	8	(13.1)			71	(28.4)

Medium	24	(39.3)			81	(32.4)

High	29	(47.5)			98	(39.2)

^a.^In the 1998 study the questionnaire included a part relating to women participating in agriculture work.

^b^Socio-economic status was defined according to farm size (low = ≤ 1ha', medium = 1.1-2.0 ha', and high = >2 ha').

**Table 2:** Agricultural works and farming descriptions of the farmers in the two study populations 1998 and 2006

	1998 (n = 61 couples)					2006 (n = 250 male farmers)
	
	Husbands		Wives			
**Characteristics**	**No**.	**(%)**	**No**.	**(%)**	**No**.	**(%)**

**Years working in agriculture**						

0-14	14	(23.0)	32	(53.0)	40	(16.0)

15-29	19	(31.0)	20	(33.0)	96	(38.4)

>29	28	(46.0)	9	(15.0)	114	(45.6)

Mean (SD)	26	(13.0)	16	(11.3)	29	(14.0)

**Farming tasks**						

Farming/cultivating	58	(95.1)	33	(54.0)	250	(100.0)

Pruning	60	(98.0)	16	(26.0)	247	(98.8)

Applying pesticides	61	(100.0)	51	(84.0)	247	(98.8)

Harvesting	61	(100.0)	56	(92.0)	250	(100.0)

Irrigating	35	(57.4)	25	(41.0)	247	(98.8)

**Types of crops grown in the farm**						

Grapes	60	(98.0)			250	(100.0)

Plums	57	(94.0)			209	(84.0)

Apples	19	(21.0)			47	(19.0)

Vegetables	27	(44.0)			198	(79.0)

Others	18	(31.0)			119	(48.0)

**Years using pesticides**						

1-10	11	(18.0)			53	(21.2)

11-20	19	(31.0)			110	(44.0)

>20	31	(51.0)			87	(35.0)

Mean (SD)	24	(12.0)			21	(11.0)

**Total pesticide units applied annually**						

≤ 40 units	26	(42.6)			239	(95.6)

>40 units	35	(57.4)			11	(4.4)

Mean (SD)	20.7	(20.8)			14.2	(20.1)

**Number of annual pesticide applications**						

≤ 8 times	51	(83.6)			214	(85.6)

>8 times	10	(16.4)			36	(14.4)

**Total hours in applying pesticides/year **^**a**^						

1-40 hours	20	(33.0)			99	(39.6)

41-80 hours	25	(41.0)			66	(26.4)

>80 hours	16	(26.0)			85	(34.0)

Mean (SD)	66	(39.0)			89	(103.0)

**Application equipment**						

Backpack sprayer	3	(5.0)			5	(2.0)

Open tractor	58	(95.0)			245	(98.0)


^a^The average working hours in applying pesticides was 8-72 hours/year for 93.5% of 1998 farmers' wives.

**Table 3:** Commonly used pesticides by farmers in Beit-U'mmar village 1998 and 2006 and their WHO classification

Pesticides	Generic name	Trade name (s)	Chemical Family	**WHO classification**^**a**^	**Use in 1998**^**b**^	**Use in 2006**^**b**^	**Remarks**^**c**^
**Insecticides**	Cyfluthrin	Baythroid	Pyrethroid	II	+	+	

	Azinphosmethyl	Cotnion*	Organophosphate	Ib	+	+	Banned

	Cypermethrin	Cympush*, Sherpaz, Siperin	Pyrethroid	II	+	+	Banned

	Diazinon	Dizictol	Organophosphate	II	+	-	

	Chlorpyriphos	Dursban, Dorsan, Pyrinex	Organophosphate	II	+	+	

	Dichlorvos	Divipan*	Organophosphate	Ib	+	+	Banned

	Parathion	Folidol*	Organophosphate	Ia	+	-	Banned

	Lambda cyhalothrin	Karate	Pyrethroid	II	+	-	

	Carbosulfan	Marshall	Carbamate	II	+	+	

	Oxydemethon methyl	Metasystox	Organophosphate	Ib	+	-	

	Dimethoate	Rogor*	Organophosphate	II	+	+	Banned

	Fenpropathrin	Smash	Pyrethroid	II	+	-	

	Methidathion	Superacide	Organophosphate	Ib	+	-	

	Methamidophos	Tamaron, Prodex	Organophosphate	Ib	+	+	

	Endosulfan	Thionex	Organochlorine	II	+	-	

	Imidacloprid	Gaucho, Confidor	Neonicotinoid	II	+	+	

	Malathion	Malathion	Organophosphate	III	+	-	

	Methiocarb	Mesurol	Carbamate	Ib	+	-	

	Maneb/manganese	Manbegan	Dithiocarbamate	U	+	-	Banned

	Abamectin	Vertimec	Biopesticide		+	-	

	Propineb	Antracol	Dithiocarbamate	U	+	+	

	DDT	Gesarol	Organochlorine	II	+	-	Banned

	Lindane	Gammacide	Organochlorine	II	+	-	Banned

	Sulphur compounds	Sulphur	Inorganic substance	U	+	+	

	Methomyl	Lannate	Carbamate	Ib	+	-	

	Fenithrothion	Fenitex	Organophosphate	II	+	-	

	Fenvalerate	Mustang	Pyrethroid	II	+	-	Banned

**Fungicides**	Triadmenol	Bayfidan*	Triazole	III	+	+	Banned

	Benomyl	Benlate	Benzimidazole	U	+	-	Banned

	Propamocarb HCL	Dynone	Carbamate	U	+	-	

	Hexaconazole	Anvil	Triazole	U	+	-	Banned

	Metalaxyl	Ridomil	Phenylamide	III	+	-	

	Penconazole	Ofir	Triazole	U	+	+	

	Myclobutanil	Systhane	Triazole	III	+	-	

	Sulphur	Gafebric	Inorganic substance	U	+	+	

	Fenarimol	Rubigan	Pyrimidine	U	+	+	

	Propiconazole	Tilit	Triazole	II	+	-	Banned

	Captan	Merpan	Phthalimide	U	+	-	Banned

	Triforine	Saparol	Piperazine	U	+	-	

	Zineb	Zidan	Dithiocarbamate	U	+	-	Banned

	Fosetyl aluminium	Aliette	Organophosphate	U	+	-	Banned

**Herbicides**	Glyphosate	Roundup	Phosphonoglycine	U	+	-	

	2,4-D	Albarsuper	Alkylchlorophenoxy	II	+	-	

	Paraquate	Dukatalon, Katalon	Bipyridylium	II	+	-	

**Rodenticides**	Ametraz	Taktic, Racumin	Amidine	III	+	-	

**Acaricides**	Propargite	Omite	Sulfite ester	III	+	-	Banned

**Fumigant**	Methyl Bromide	Methyl Bromide		FM	+	+	

^a ^Ia, extremely hazardous; Ib, highly hazardous; II, moderately hazardous; III, slightly hazardous; U, unlikely to present acute hazard in normal use; FM, fumigant (WHO,2005)

^b^+ means yes in use, ―means not in use

^c^Banned according to references (18, 20) and reference 21 (Annex3, Rotterdam Convention on the Prior Informed Consent Procedure for Certain Hazardous Chemicals and Pesticides in International Trade, Rotterdam, 10 September 1998).

**Table 4:** Handling and practicing of pesticides in the two study populations, Beit-U'mmar village, 1998 and 2006

Item	1998 (n = 61)		2006 (n = 250)		Crude Prevalence Difference (PD)	**Adjusted Prevalence Difference (aPD)**^**a**^
			
	**No**.	(%)	**No**.	(%)		
Using the required amounts of pesticides	15	(25.0)	208	(83.0)	+0.58 (+0.47, +0.69)	+0.57(+0.48,+0.68)*

Preparing (mixing) pesticides at home	44	(72.0)	192	(77.0)	+0.05 (- 0.07, +0.17)	+0.03(-0.10, +0.16)

Storing pesticides at home	51	(84.0)	196	(78.0)	-0.06 (- 0.17, +0.06)	-0.01(-0.13, +0.11)

Washing working clothes with the family clothes	51	(84.0)	236	(94.0)	+0.10 (+0.03,+0.18)	+0.10 (+0.02,+0.19)*

Cleaning spraying equipment after work	37	(60.7)	189	(75.6)	+0.15 (+0.02, +0.27)	+0.16 (+0.03, +0.29)*

Taking a meal at work place	40	(65.6)	71	(28.4)	-0.37 (-0.50, -0.24)	-0.33 (-0.47, -0.19)*

Smoking while applying pesticides	29	(47.5)	64	(25.6)	-0.22 (-0.36, -0.09)	-0.20 (-0.34, -0.07)*

Considering the safety period	6	(9.8)	244	(97.6)	+0.88 (+0.82, +0.93)	+0.89 (+0.83, +0.95)*

Using more than 40 units of pesticides/year	35	(57.0)	11	(4.4)	-0.53 (-0.61, -0.45)	-0.51 (-0.60, -0.43)*

Applying pesticides more than eight times/year	10	(16.4)	36	(14.4)	-0.02 (-0.12, +0.08)	-0.02 (-0.13, +0.09)

^a ^Adjusted for farmer's age, years of farming, educational years, socio-economic status, and main crops (plums, apples, vegetables)

*Statistically significance p ≤ 0.05

**Table 5:** Use of personal protective equipment (PPE) by study population, Beit-U'mmar village, 1998 and 2006

PPE	1998 (n = 61)		2006 (n = 250)		Crude Prevalence Difference (PD)	**Adjusted Prevalence Difference (aPD)**^**a**^
			
	**No**.	(%)	**No**.	(%)		
Gloves	15	(25.0)	73	(29.0)	+0.05 (-0.08, +0.17)	+0.04 (-0.10, +0.17)

Mask	9	(15.0)	73	(29.0)	+0.14 (+0.02, +0.27)	+0.11 (-0.02, +0.25)

Goggles	8	(13.0)	66	(26.0)	+0.13 (+0.01, +0.25)	+0.12 (-0.01, +0.25)

Long boots	11	(18.0)	70	(28.0)	+0.10 (-0.02, +0.22)	+0.07 (-0.07,+0.20)

Coverall	15	(25.0)	71	(28.0)	+0.04 (-0.09, +0.16)	+0.01 (-0.13,+0.14)

^a ^Adjusted for farmer's age, years of farming, educational years, socio-economic status, and main crops (plums, apples, vegetable)

### Data Collection

In 1998, the principal investigator (YI) filled out the questionnaire through face-to-face interviews with male farmers, while a trained female health worker from the village interviewed their wives. Also in 1998 and before conducting the interviews with the farmers we conducted an in-depth interview with a focus group to get a more comprehensive description of the situation in the village. In the 2006 study, two trained health workers from the village did the structured face-to-face interviews with male farmers. In 1998, data on compounds and quantities of pesticides used were collected both from the pesticide distributor in the village (i.e., to get a more complete description of the agricultural situation in the village) and from the farmers themselves. In 2006, information on compounds and quantities of pesticides was collected solely from the farmers.

### Statistical analyses

In order to calculate associations we used ordinary generalized linear model (GLM) regression, estimating additive differences in prevalences (PD) with corresponding 95% confidence intervals (CI). In regression analyses, we controlled for: farmer's age, years of farming, educational years, socio-economic status, and main crops grown on the farm (plums, apples, vegetables, but not grapes because only one farmer did not produce grapes).

We applied the same categories in the analysis as those presented in Table 1 and 2. In all analyses, the 1998 prevalence served as a reference. Thus, negative PDs indicate reductions and positive PDs indicate increases between 1998 and 2006. We used SPSS (Statistical Package for Social Sciences, version 16) and STATA (version10) for data analyses, and the level of significance was set to 95% CI or p ≤ 0.05.

## Results

Farmers were slightly older in the 2006 study than in 1998 and were less likely to smoke (36.4% vs. 80.3%). The two populations were comparable concerning educational attainment but the 2006 sample had a larger proportion of low socio-economic subjects (Table1).

Table 2 shows that the farmers in 1998 had been working in agriculture longer than the farmers in 2006. Both farmers from 1998 and 2006 used open tractors and backpacks when spraying pesticides on the crops. In 1998, we found a high average usage of pesticides for vegetables (3 units/1000 sq.m), and for plums and grapes (2 units/1000 sq.m) (data not shown). The highest mean number of applications per season was for grapes (7 times/season), and for plums and vegetables (3 times/season). In 1998, wives participated in all farming tasks, including applying pesticides. Furthermore, farmers in 1998 used more pesticides compared with 2006, with averages of 21.0 and 14.0 units, respectively.

In the 1998 study, there were 47 pesticide compounds reported whereas only 16 compounds were reported in 2006. Only five banned compounds out of 17 used in 1998 were still in use in 2006. Seven highly hazardous compounds (Class Ib) were used in 1998 and only three in 2006. This tendency was also seen for moderately hazardous products (Class II), of which 18 were used in 1998 and six were used in 2006 (Table 3).

Table 4 shows that an improvement might have taken place in pesticide handling and practices in 2006 compared to 1998. There was considerable improvement in adhering to the safety period for re-entering a sprayed field. Also, 2006 farmers reported that they used required pesticide amounts and were less likely to smoke and eat in the fields. However, washing working clothes and family clothes together was slightly more common in 2006.

Personal protective equipment (PPE) was rarely used by the farmers in both studies, but there was a slight improvement in 2006 mainly in wearing mask and goggles (Table 5). However, all PDs were non-significant. The reasons for not using PPE were due to discomfort from hot weather and that it hampered work. Further some farmers stated that PPE was unnecessary, costly, or unavailable. The majority of the 1998 farmers reported that they believed immunity from pesticides could be developed over time (this item was not addressed in 2006).

## Discussion

Some positive changes were found over time in the handling of pesticides. These included less use of large quantities of pesticides, improved complying with the recommended dosage of pesticide application, and following the recommended safety period. Farmers were also less likely to smoke while applying pesticides and eat at the work place. However, there were still problems in 2006 regarding pesticide storage, farmers' habits after applying pesticides as washing working clothes with the family clothes, and the use of some highly hazardous pesticides.

Design-wise, these two studies are cross-sectional. There are some strengths: Both study questionnaires were piloted and thereafter modified, and all interviews were performed after initial training. There are also several weaknesses that could influence the results. The two studies were initiated for other reasons than measuring changes in pesticide use and practices. The inclusion criteria for participation were somewhat different. The 1998 sample was hampered by low response and added uncertainty because there was no official registry of farmers in the village in 1998. This endangers the assumption that both the 1998 and the 2006 sample should be representative of all farmers in the village. Accordingly, the apparent improvement over time could be due to selection bias in the case 1998 non-participation was selective, being higher among farmers who had restricted use of pesticides or who had sound pesticide use practices.

The data were mostly based on identical questionnaires in both years, but there were some comparability problems. In 1998, data on pesticide compounds and quantities were collected from farmers and the pesticide distributor in the village, while 2006 data was collected from the farmers only. In 1998, farmers were asked about the names of pesticides used for each type of crops that they cultivated whereas 2006 farmers were asked about the names of pesticides used in each month of the year. Potential underestimation of pesticide use in 2006 could result in a false impression of improvement over time, in particular regarding the compounds applied.

Despite apparent positive changes in the handling and practising of pesticides, Palestinian farmers are still at risk of the adverse effects of pesticide exposure. Farmers in the present study had used pesticides for a long time, making them more at risk of cumulative exposure, in accordance with Del Prado-Lu [15]. Farmers reported smoking while applying pesticides and also having a meal in the field. This increases their risk of exposure to pesticides, which is confirmed in other studies from oPt and other developing countries [25,27-30]. Another exposure opportunity is using an open tractor for spraying pesticides. This could lead to higher exposure as well as contamination of the environment and the general population, also this in accordance with studies from oPt and other developing countries [15,20,27].

We found moderate but non-significant positive changes in the use of PPE in 2006. This might not be sufficient to reduce adverse effects of pesticide exposure. Farmers in both studies explained non-compliance by stating that PPE caused discomfort on hot days and hampers work. Further, some farmers stated that PPE is unnecessary, costly or unavailable, in line with previous studies [25,27,28,30-34]. Rare use of PPE was reported in several studies among Palestinian farmers [20,22,25] and among other farmers in developing [15,27-38] and developed countries [39,40]. Richter and co-workers found that using protective measures was poor among West Bank farmers [31]. Furthermore, it was suggested that the efficacy of whole day use of PPE might be exaggerated, and that the most important factors are substitution and reduction in using pesticides in order to reduce exposure [41]. In addition, Richter and co-workers [41] found that a reduction in pesticide (organophosphate) use resulted in increased crop (cotton) yields. The reported belief that 1998 users develop immunity from pesticides has also been reported in other studies in oPt [20,22,25,42].

In the present study we found that the farmers' family members, including children, could be exposed to pesticides indirectly due to the take-home exposure or para-occupational exposure pathways, which include returning home with working clothes, washing and cleaning contaminated clothes in the home laundry, mixing and storing pesticides at home, and cleaning equipments used for application at home. These findings were also consistent with other studies conducted among Palestinian farmers and in other parts of the world [22,25,42-47].

Farmer families residing in the vicinity of the orchards is another possible reason of exposure. Several studies have found that farm homes have a greater frequency of detectable residues of pesticides and have higher concentrations of pesticides in dust than in reference homes, potentially leading to greater exposure to pesticides among family members [43-49]. Richter and co-workers [41] found that children living in houses close to treated farmland may have a greater potential exposure if pesticides drift into residential or other areas in which children are playing.

The 1998 participants reported more extensive use (mean 20.7 units) in 1998 compared to 2006 (mean 14.2 units). This reduction was supported by regional statistics [23] as well as by the local pesticide distributor, who assessed the total sales amounts to be considerable lower in more recent years compared to 1998 (personal information, 2010).

We should have the potential validity problems in mind when making inferences. With this caveat, the findings suggest positive changes in handling and practising of pesticides by farmers in Beit-U'mmar village. These changes could have several explanations. Various interventions related to pesticide management and monitoring have been implemented by the Palestinian National Authority (PNA) and other NGOs. Firstly, a new Palestinian agricultural law was enacted that contained a chapter on pesticide management (i.e., Palestinian Agricultural Law, 2003). According to this law, manufacturing, importing, preparing, producing, distributing, selling, and storing of pesticides should be under the control and permission of the Ministry of Agriculture. This law stipulates that pesticide containers should be labelled and that all labels should contain all required information in Arabic, including the degree of toxicity, safety precautions, and active ingredients. Secondly, the Ministry of Agriculture carried out awareness-raising activities in coordination with NGOs in all districts of the West Bank in addition to those activities conducted by Hebron University. These activities included holding workshops and distributing leaflets and posters, which were aimed to increase knowledge and awareness among farmers and pesticide distributors about the safe use of pesticides. Thirdly, the Ministry of Agriculture sent agricultural engineers to each municipality in the West Bank to provide guidance and extension services on a variety of topics, including pesticide use and handling [Hebron Agriculture Department, 2006 personal communication]. The training and educational interventions conducted between 1998 and 2006 could possibly explain part of the observed reductions in pesticide usage and number of compounds as well as positive changes in handling and dealing with pesticides.

Furthermore, an integrated pest management system between Israel, Palestine, and Jordan was established to decrease pesticide use, promote integrated pest management, and restrict ecosystem damage while maintaining or increasing cotton yield. Preliminary results of this program indicate that it has been successful [31,50]. Moreover, the PNA has helped minimize the trade of contraband pesticides. While in 1998, there was free movement of goods, the restrictions and closures associated with the second Intifada in 2000 led to a marked increase in the number of illegal pesticides. Since the end of the Intifada, however, the laws and regulations with respect to pesticides were once again enforced, decreasing the number of illegal pesticides on the market. In addition to the above factors for reduction in pesticides compounds and usage is the political situation and the conflict their. During the second Intifada (after 2000), several chemicals including some pesticides such as sulphur compounds thionex, lannate, urea, and other compounds containing ammonia and nitrate became prohibited in the Palestinian areas for what is called Israeli security reasons [Hebron Agricultural Department, personal communication, 2010]. Another potential explanation of the changes between 1998 and 2006 could be climatic variations. Weather and climate affect many agricultural decisions including crop choices, water management, and crop protection [51]. Koleva and co-workers [51] and Chen and McCarl [52] found that weather variability and climate change was associated with a decline in pesticide use in the US. In recent years there has been a climatic variation which lead to a decline in rainfall in oPt [Palestinian Meteorological Authority Statistics, personal communication, 2010], which might have lead to changes in cultivation and pesticide use.

## Conclusions

Exposure to pesticides among farmers and farmers' families is a major health threat. This well known fact is one obvious and important rational for safe handling and practices of pesticides. The present study suggest that farmers, their family members, and perhaps the entire population of Beit-U'mmer village are both directly and indirectly exposed to highly hazardous, restricted, and banned pesticides, with insufficient protection. Educational and training interventions on pesticide handling and safety precautions are recommended to change this situation. In addition, governmental interventions and efforts, such as restrictions on hazardous pesticides, monitoring of labels, and enforcement of good agricultural practices are needed to decrease pesticide exposure of farmers and the general population.

## List of Abbreviations

GNP: Gross National Products; PPE: Personal Protective Equipment; ha': hectare; GLM: Generalized Linear Model; CI: Confidence Interval; PD: Prevalence difference; PNA: Palestinian National Authority; oPt; occupied Palestinian territory; NGO: Non-governmental Organization.

## Competing interests

The authors declare that they have no competing interests. The authors YI and FK have on several occasions been invited by different municipalities in Hebron district, the Independent Palestinian Committee for Drug Studies, and by the University of Hebron to inform about pesticide use and their adverse effects. The target groups for these presentations were local farmers, women, and students.

## Authors' contributions

YI, KN, EB, and PK designed both studies while FK designed the 2006 study. YI and FK participated in data collection and data collection monitoring. YI prepared the datasets, wrote the first draft of the manuscript and performed the preliminary statistical analyses. YI, KN, and PK participated in data analyses. YI, KN, FK, EB and PK participated in the interpretations of the results. All authors participated in the conceptualisation and writing of the paper, and have seen, reviewed and approved the final version.
